# Beyond family donors: building reliable emergency blood access in Somalia

**DOI:** 10.1016/j.afjem.2026.101007

**Published:** 2026-09-08

**Authors:** Abdullahi Abdi Yusuf, Deka Ahmed Abdi, Aden Mohamed Hirsi, Ayan Mohamed Hassan, Khalid Hussein Ulusow

**Affiliations:** a Faculty of Medicine and Surgery, Somali National University, Mogadishu, Somalia; b BENAADIR Research, Consultancy and Evaluation Center, Mogadishu, Somalia; c Faculty of Medicine & surgery, Salaam university, Mogadishu, Somalia

**Keywords:** Blood transfusion, Emergency care, Family donors, Voluntary blood donation, Blood availability, Somalia

## Abstract

• Dependence on family or replacement donors can make access to emergency transfusion reactive rather than reliably available at the point of care, a problem directly relevant to trauma, obstetric haemorrhage, and urgent surgery in resource-constrained African settings.
• Somalia-specific evidence shows that family donors can dominate local blood supply and that emergency blood transfusion services remain limited across surveyed hospitals, making reliable blood access an important emergency-care systems issue.
• The correspondence proposes a practical shift from patient-triggered donor mobilisation toward regular voluntary donation and system readiness, with lessons relevant to Somalia and other African settings facing constrained blood-service capacity.

Prompt availability of safe and compatible blood is critical to effective emergency care, especially in cases of major hemorrhage, obstetric emergencies, trauma and urgent surgery. But when transfusion depends on mobilizing relatives or friends after the patient presents, access depends on social networks rather than on system readiness. The World Health Organization (WHO) recommends national blood systems based on regular voluntary, unpaid donation as this provides the most reliable and safest donor base [1]. The policy question for Somalia is therefore not whether family donors should disappear, but whether emergency care should continue to rely on them.

Short-term gaps are filled by family or replacement donation but it is intrinsically reactive [2]. In a time-critical emergency, blood cannot be considered reliably available if donor identification, eligibility screening, infectious-disease testing, blood grouping, compatibility assessment, and collection are performed only after a transfusion is needed. Experiences from low resource settings show that access to emergency blood can be limited by weaknesses throughout the transfusion chain, including supply, testing, transport, and communication [2,3]. It is important to make this distinction, as donation is not equivalent to availability and availability is not equivalent to emergency transfusion readiness.

However, Somalia-specific evidence gives the concern practical relevance. At SOS Hospital Mogadishu, 36,296 donations were recorded between 2016 and 2022, out of which 80.8% were family donors [4]. This hospital-based finding should not be generalized as a national estimate, but it demonstrates that replacement donation can dominate local blood supply. More generally, a national survey of 131 Somali hospitals revealed that only 9.2% of them had emergency blood transfusion services [5]. In sum, these results suggest that the key challenge is not mere donor recruitment but the presence of screened and compatible blood at the locations of emergencies.

Somalia should then gradually shift away from patient-triggered replacement donation to a more predictable emergency blood system. These priorities include the need to increase regular voluntary non-remunerated donation, maintain blood stocks that are appropriately managed, quality-assured screening for HIV, hepatitis B, hepatitis C and syphilis, and strengthening blood grouping, compatibility testing, cold-chain systems, inventory management, haemovigilance and communication between blood banks and hospitals [1,2]. Also important are transport and referral pathways. African data show that blood may be out of stock at the bedside despite an organized blood service when supply, transport, sample processing, protocols or communication break down [3].

The final measure of emergency blood readiness is not the number of donors recruited or units collected, but safe, compatible blood available to patients within a clinically meaningful time. Somalia needs to go beyond family donors as the default emergency solution and create a system where blood is available as the health system is prepared before the emergency strikes.

## Funding

This work received no external funding.

## Ethical approval

Ethical approval was not required because this article did not involve human participants or primary data collection.

## Data availability statement

No new data were generated or analyzed for this article.

## Declaration of generative AI Use

During manuscript preparation, the authors used ChatGPT (OpenAI) solely for language editing and refinement. All content was reviewed and approved by the authors, who take full responsibility for the final manuscript.

## Dissemination of results

This correspondence is based on published evidence and does not report primary research involving study participants or study sites. Accordingly, there are no participant-specific results requiring return. The article’s conclusions and policy recommendations will be disseminated through publication in the African Journal of Emergency Medicine and shared with relevant Somali emergency-care clinicians, blood-service stakeholders, academic institutions, and health-system decision-makers to support discussion on reliable emergency blood access

## CRediT authorship contribution statement

**Abdullahi Abdi Yusuf:** Conceptualization, Investigation, Writing – original draft, Writing – review & editing, Project administration. **Deka Ahmed Abdi:** Investigation, Writing – review & editing. **Aden Mohamed Hirsi:** Investigation, Writing – review & editing. **Ayan Mohamed Hassan:** Investigation, Writing – review & editing. **Khalid Hussein Ulusow:** Investigation, Writing – review & editing.

## Declaration of competing interest

The authors declare no competing interests.
